# Curative effect analysis of robot-assisted drainage surgery in treatment of spontaneous hypertensive brainstem hemorrhage

**DOI:** 10.3389/fneur.2024.1352949

**Published:** 2024-02-26

**Authors:** Zhiji Tang, Weilong Huang, Qiqi Chen, Changgui Guo, Kuan Zheng, Wenjin Wei, Qiuhua Jiang, Ruijin Yang

**Affiliations:** ^1^Department of Neurosurgery, Ganzhou People’s Hospital, Ganzhou, Jiangxi, China; ^2^Department of Magnetoencephalography, Nanjing Brain Hospital Affiliated to Nanjing Medical University, Nanjing, China

**Keywords:** spontaneous hypertensive brainstem hemorrhage, surgical treatment, robot-assisted drainage, stereotactic system, prognosis

## Abstract

**Objective:**

Spontaneous hypertensive brainstem hemorrhage (HBSH) is characterized by sudden onset, rapid progression and poor prognosis. There has been a growing tendency of surgical treatment for HBSH. This study aimed to investigate outcomes and potential factors associated with the prognosis of robot-assisted drainage surgery for HBSH treatment.

**Methods:**

Patients with HBSH from July 2016 to March 2023 at a single neurosurgery center were included and divided into conservative group and surgical groups. Baseline and clinical data, radiographic characteristics, complications, and outcome evaluations were recorded and analyzed.

**Results:**

A total of 125 patients, with 74 in the conservative group and 51 in the surgical group, were enrolled in the study. Mortality at 6 months was 59/74 (79.7%) in the conservative group and 9/51 (17.6%) in the surgical group. Twenty-four patients (47.1%) achieved favorable outcomes in the surgical group, whereas this rate in the conservative group was only 5.4% (4/74). There was a significant difference in NIHSS, GCS, and mRS at 6 months between surviving patients in the conservative and surgical groups. In prognostic analysis in the surgical subgroup, initial GCS score [5 (IQR 4–7) vs. 3 (IQR 3–4), *p* < 0.001], NIHSS [36 (IQR 32–38) vs. 40 (IQR 38–40), *p* < 0.001], smoking history [45.8% (11/24) vs. 74.1% (20/27), *p* = 0.039], hematoma volume [6.9 (IQR 6.2–7.6) vs. 9.6 (IQR 7.3–11.4), *p* = 0.001], and hemorrhage location (*p* = 0.001) were potential risk factors for poor 6-month prognosis after robot-assisted surgery for HBSH.

**Conclusion:**

Based on the results of this study, robot-assisted minimally invasive drainage of brain stem hematoma may significantly reduce mortality and improve prognosis. Surgery should be conducted for selected patients.

## Introduction

Spontaneous hypertensive brainstem hemorrhage (HBSH) is one of the most serious cerebrovascular diseases due to its sudden onset, rapid progression and poor prognosis and is generally accompanied by high disability and fatality rates (1). The treatments, rehabilitation therapy, and permanent sequelae associated with HBSH impose a substantial economic burden on patients’ families and have profound impacts on society. The incidence of HBSH is approximately 2 to 4 per year in 100,000 people (2, 3), and it is thus generally considered uncommon (4). In one study, the incidence of brain stem bleeding accounted for only 5.8% of all intracerebral hemorrhage (ICH) cases. However, the incidence of HBSH is approximately two times higher in East Asia than in other regions (5–7), and its etiology remains unclear. Currently, most neurologists still advocate conservative procedures for HBSH. However, conservative management still carries a high mortality and morbidity rate. Some studies revealed that the mortality rate of HBSH the mortality rates vary widely from 40 to 90% with conservative management (8–10). Some surgeons even believe that surgery is ineffective for HBSH patients with irreversible coma because of a disastrous outcome (11–13). However, there has been a growing tendency of surgical treatment for HBSH in China. Among such reports, microsurgery and stereotactic drainage are mentioned most (14–18). With the development of high-end level microscopy, neuronavigation and real-time electrophysiological monitoring technology, microsurgery has played an important role in microsurgical treatment for HBSH (10, 19). Some encouraging outcomes by performing surgical management via the lateral or midline suboccipital and trans-rhomboid fossa approach have been reported in the literature (18, 20). However, microsurgery is characterized by potential iatrogenic damage, postoperative reactions, and a high level of surgical difficulty. Consequently, it is necessary to explore surgical treatments that are less invasive and less destructive to the brain stem (21).

Stereotactics have the function of accurate three-dimensional spatial positioning such that the surgeon can accurately reach the millimeter level of anatomy or lesion location in the brain through only a small channel. Therefore, minimally invasive stereotactic surgery has been applied for brainstem hemorrhage surgery, with encouraging results since 1989 (6, 17, 22, 23). As an accurate auxiliary tool for surgical location, the robot stereotactic system is used in intracerebral hematoma puncture in addition to stereoelectroencephalogra-phy (SEEG) implantation and deep-brain stimulation (DBS) (24–26). Compared with frame-based stereotactic procedures, robot-assisted technology can reach some targets that frame-based stereotactic surgery cannot, reduce the operation time and prevent the pain caused by installing the frame (27).

At present, there are few reports regarding robot-assisted technology for treatment of HBSH. Thus, we report this retrospective study aiming to investigate the outcomes and potential factors associated with the prognosis of robot-assisted drainage surgery for HBSH treatment.

## Methods

### Patient population

This retrospective, observational study was performed from July 2016 to March 2023 in a single neurosurgery center that serves a population of approximately 9 million and has more than 160 beds, including a neurosurgical intensive care unit (22 beds) at Ganzhou People’s Hospital, Jiangxi Province, China. During this period, a total of 252 patients with spontaneous HBSH were admitted to our department.

### Inclusion and exclusion criteria

To observe the difference between conservative management and surgical treatment of HBSH, our inclusion criterion was patients with a Glasgow Coma Scale (GCS) score < = 8 on admission. Our exclusion criteria were as follows: (1) suspected cavernous hemangioma hemorrhage on imaging; (2) hemorrhage caused by aneurysm rupture or arteriovenous malformation; (3) respiratory and circulatory failure needing curative care; and (4) suspected history of brainstem trauma or tumor; (5) brainstem hemorrhage combined with significant thalamic or basal ganglia hemorrhage.

### Conservative and surgical treatment

All HBSH patients were admitted to our neurosurgical intensive care unit, and initial management and resuscitation of all the patients were performed. Those who were eligible for inclusion were offered the option of surgery. We discussed in detail with the patient’s family the potential risks and benefits of medication and surgery. The patients’ relatives decided whether to agree with conservative treatment or surgery. Once the decision was made, the patients were divided into a conservative group and a surgical group. Conservative therapeutic protocols were routinely administered for the conservative group, including elevation of the head of the bed, lowering of systolic blood pressure (SBP), sedation, tracheal intubation/mechanical ventilation, hyperosmolar treatment, and hypothermia, among others (28).

**Figure 1:**
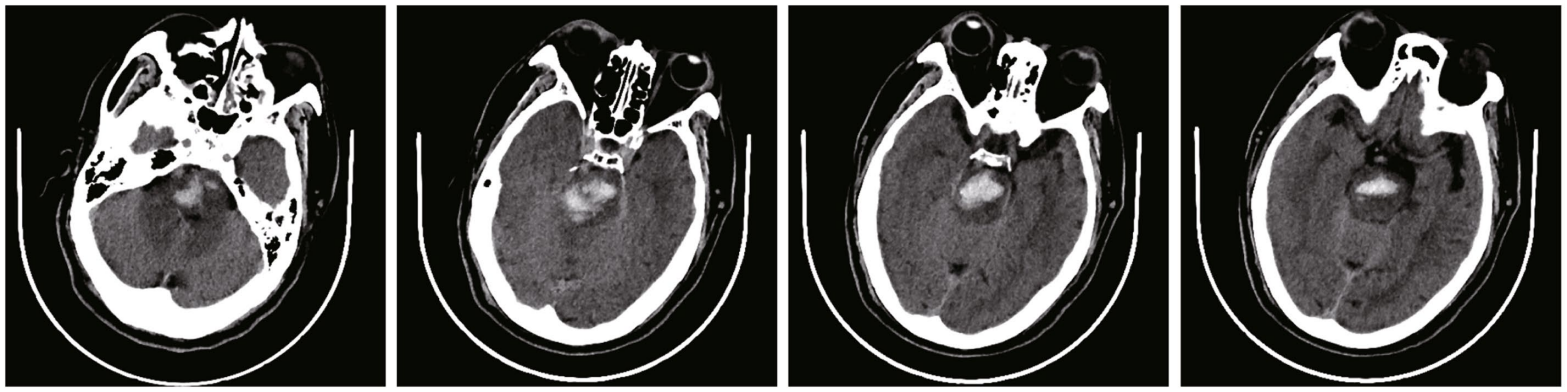
Pre-operative CT scans of a 61 year-old male patients at admission, with a pontine hemorrhage of 7.9 mL (The images in this manuscript were all from the same patient).

**Figure 2:**
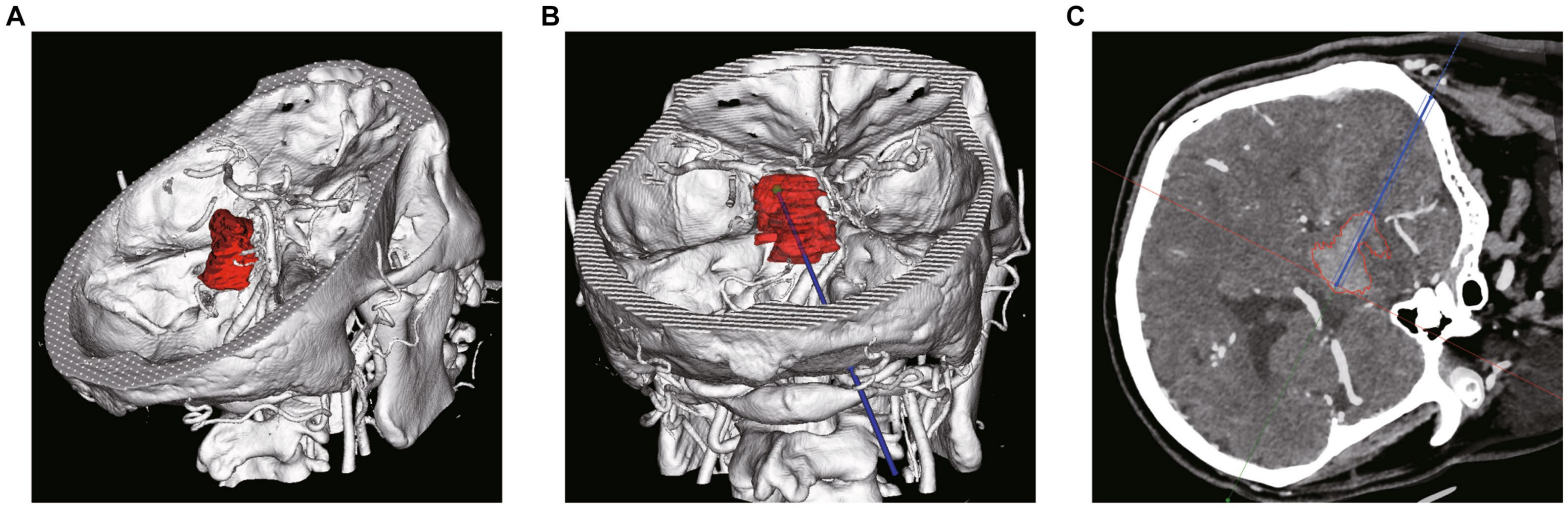
Pre-operative plan using robot planning system. **(A)** 3D shape and location reconstruction of hematoma. **(B)** The target and drainage path was planned through the long axis of the hematoma. **(C)** Avoiding the dentate gyrus and blood vessels on the basis of CTA and CT image fusion.

**Figure 3:**
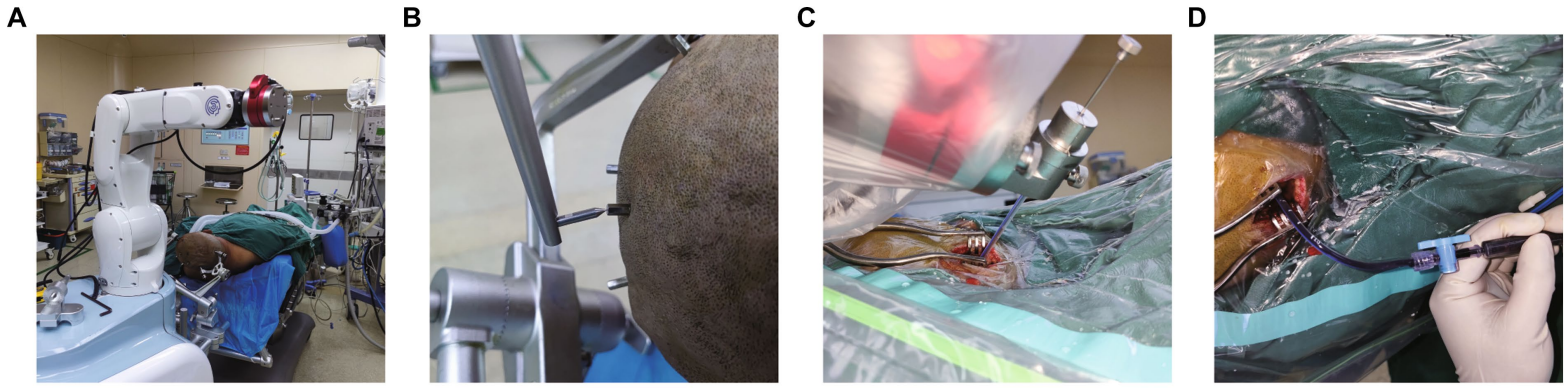
Robot-assisted Drainage surgical process. **(A)** Patient’s head is firmly connected to the robot. **(B)** Metal markers were firmly inserted into the patient’s head to adopt the minimum registration error method. **(C)** Inserting the puncture tube slowly under the guidance of robotic arm. **(D)** Intraoperative suction of the partially liquefied hematoma.

**Figure 4:**
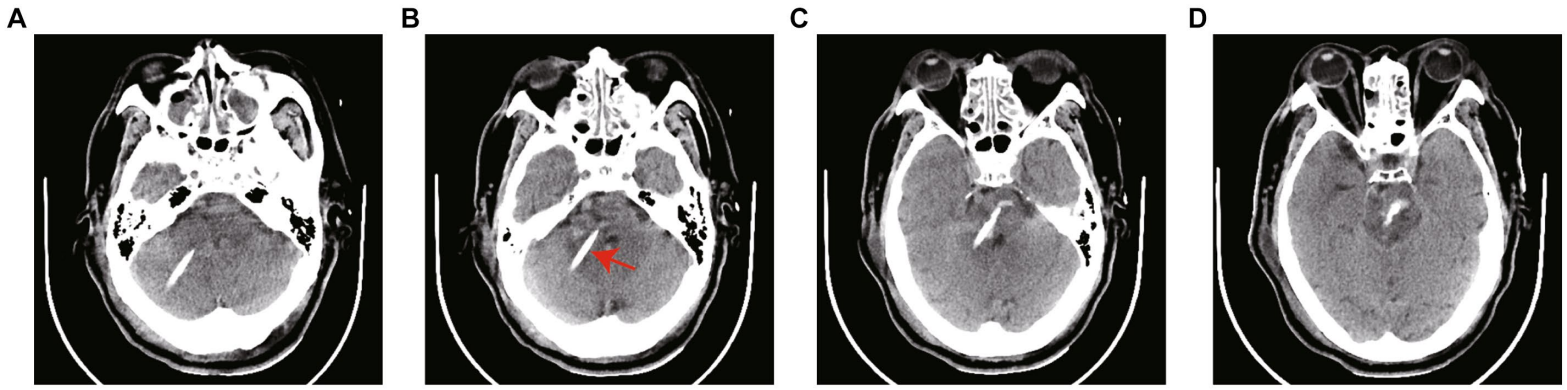
**(A–D)** CT scans 12 h after surgery. The arrow in the figure refers to the drainage tube. The hematoma was dissolved by injecting 20,000–30,000 IU of urokinase into the hematoma cavity once a day. The drainage tube was removed according to the results of CT scanning.

**Figure 5:**
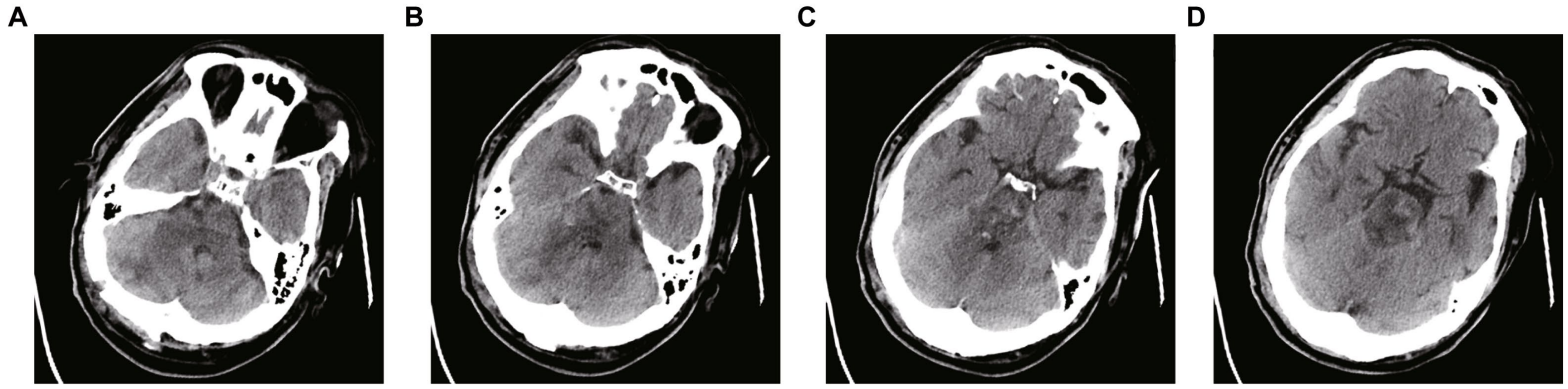
**(A–D)** The follow-up CT scans 70 h after surgery showed the hematoma was nearly completely removed and the drainage tube had been extracted.

The surgical group was treated with robot-assisted drainage of the brain stem hematoma. Our center uses the Sinovation SR1 robot (Sinovation, Beijing, China) to perform these operations. In addition, this model of robot is the first neurosurgery robot that has passed the national innovation review in China. The robot-assisted surgical procedure was administered as follows. Before the operation, 5 to 6 cranial markers were firmly inserted into the patient’s head, then extremely thin-layer CT and CTA scanning (layer space 0.625 mm) were performed, and Dicom format data were obtained. After loading the data into the Sinovation SR1 software system, we conducted image fusion and 3D reconstruction of the skull, hematoma and vessels. The best puncture path was discussed and determined by at least 2 well-experienced neurosurgeons. The target and drainage path was individually planned depending on the 3D shape and location of the hematoma; it was usually based on the principle of which the drainage path passes through the long axis of the hematoma. Avoiding the dentate gyrus and blood vessels was also essential in the preoperative projection. Prior to the operation, a Fisher head frame was applied to fix the patient’s head and firmly connected to the robot, ensuring that they were in a relatively stationary status. Before cutting the scalp, one of the metal markers was used as a verification point to validate that the registered error of the robotic arm was less than 0.3 mm. After the initial phase of the operation, the robotic arm is automatically positioned on the preset path, the guidance of which determines the surgical incision. After cutting a tiny incision on the scalp, a 3 mm diameter hole was drilled in the skull. In terms of the preoperative surgical plan, the robotic arm was moved to the preset position. Then, after locking the robotic arm, a No. 12 silicone puncture tube with a stylet inside was slowly inserted into the hematoma cavity through the limiting device on the end of the robotic arm. Postoperative CT scanning was immediately conducted to validate whether the drainage tube had been placed in the ideal position. After the operation, the hematoma was dissolved by injecting 20,000–30,000 IU of urokinase into the hematoma cavity once a day. In general, we removed the drainage tube according to the results of CT scanning 1–3 days after the surgery (Figures 1–5).

### Data collection and definition

Baseline data were extracted from hospital electronic medical records, including sex, age, SBP and DBP on admission, history of smoking and underlying diseases, and medication before brainstem hemorrhage, such as antihypersensitive, anticoagulant and antiplatelet drugs. The initial Glasgow Coma Scale (GCS) score and National Institutes of Health Stroke Scale (NIHSS) were used to evaluate the severity of neurological impairment in patients with HBSH.

Each patient’s radiographic characteristics of CT scans on admission were reviewed and recorded by at least two neurologists. The volume of the brain stem hematoma was assessed based on the Coniglobus formula, which is ABC/2, where A is the longest diameter of the hematoma, B is the vertical diameter of A, and C is the approximate thickness of the hematoma (29). Hematoma clearance rate = (postoperative volume)/(preoperative volume) × 100%. The location of brain stem hemorrhage was divided into four types according to Chung and Park’s classification method, namely, unilateral tegmental type, bilateral tegmental type, basal-tegmental type, and massive type (30). Intraventricular hemorrhage was also noted. Complications such as hyperthermia, stress ulcers, and tracheotomy rates were recorded. The time from event to operation was defined as the duration from occurrence of HBSH to performing the operation. Hematoma clearance refers to the clearance rate 72 h after surgery and is calculated according to the following formula: (1-residual hematoma/initial hematoma)*100%.

All patients had received follow-up for at least 6 months at the hospital or by telephone/video interview to their relatives. First, outcome evaluations, such as 6-month mortality, modified Rankin scale (mRS) and complications, were compared between the conservative and surgical groups. Second, for all patients surviving to 6 months, GCS score and NIHSS at 6 months were obtained to assess the extent of neurological impairment. Functional status was determined using the mRS at 6 months. Finally, specifically for the surgical subgroup, we compared baseline and clinical data in patients with favorable and unfavorable prognoses. In the present study, an unfavorable outcome was defined as an mRS score of 5 and 6, whereas a favorable outcome was defined as 0 to 4.

### Statistical analysis

Continuous data such as age, GCS, NIHSS, blood pressure, and hematoma volume are presented as medians with 25th and 75th quartiles (IQRs) due to their skewed distribution; categorical data are expressed as numbers and percentages. Comparisons were performed using the chi-square test for categorical variables and the Mann–Whitney U test for continuous variables. All statistical analyses were performed with SPSS (version 20.0, IBM SPSS Statistics).

## Results

### Patient features

Of the 252 patients with spontaneous HBSH who were treated at our institution during the study period, 125 who met the criteria were enrolled. Fifty-one patients underwent surgery; 74 were managed conservatively. The patients’ demographic data and baseline characteristics are summarized in Table 1. In total, the median age at presentation was 55 years [IQR 47–62], and most of the patients (94 (75.2%)) were male. The median initial GCS score on admission was 4 (3–5), and the median NIHSS score was 38 [IQR 36–40]. Both SBP and DBP were extremely high in all patients, with median numbers of 178 (156–198) and 101 (91–109), respectively. Regarding available medical history of all patients, 75 (60.0%) had a history of smoking, and 17 (13.6%) and 23 (18.4%) had previous diabetes and hyperlipidemia, respectively. In terms of medication before HBSH, 1 (0.8%) patient had taken anticoagulant drugs before the event, and 16 (12.8%) had a history of taking antiplatelet drugs. Among all patients, up to 102 (81.6%) had a history of irregular administration of antihypertensive drugs. As described in Table 1, there were no differences between the conservative and surgical groups regarding demographic data and baseline characteristics.

**Table 1:** Baseline characteristics of the total, conservative and surgical cohorts.

	Total (*n* = 125)	Conservative group (*n* = 74)	Surgical group (*n* = 51)	*p*
Age	55 (47–62)	54 (44.8–61)	55 (49–63)	0.222
Initial GCS score	4 (3–5)	4 (3–5)	4 (3–5)	0.763
NIHSS on admission	38 (36–40)	38 (36–40)	38 (36–40)	0.753
Sex				0.16
Male	94 (75.2%)	59 (79.7%)	35 (68.6%)	
Female	31 (24.8%)	15 (20.3%)	16 (31.4%)	
SBP on admission	178 (156–198)	180 (155–202)	172 (157–187)	0.091
DBP on admission	101 (91–109)	101 (91–112)	99 (92–107)	0.284
Medical history				
Smoking (yes)	75 (60.0%)	44 (59.5%)	31 (60.8%)	0.882
Diabetes (yes)	17 (13.6%)	10 (13.5%)	7 (13.7%)	0.973
Hyperlipidemia (yes)	23 (18.4%)	14 (18.9%)	9 (17.6%)	0.857
Medication before HBSH				
Anticoagulant drugs	1 (0.8%)	0 (0.0%)	1 (0.2%)	0.227
Antiplatelet drugs	16 (12.8%)	12 (16.2%)	4 (7.8%)	0.168
Irregular antihypertensive therapy(yes)	102 (81.6%)	62 (83.8%)	40 (78.4%)	0.449

GCS, Glasgow Score Scale; NIHSS, National Institutes of Health Stroke Scale.

### Hematoma characteristics

The characteristics of the hematomas of all patients are presented in Table 2. The median hematoma volume was 7.6 mL [IQR 6.0–9.6]. Regarding classification, the hematoma was located unilaterally in 17 patients (13.6%), bilaterally in 24 (19.2%), basally in 39 (31.2%), and massively in 45 (36%). In addition, 32 patients had intraventricular hematoma. Table 2 also shows that the characteristics of hematomas were comparable between the conservative and surgical groups. *p* values in comparisons of the hematoma volume, hematoma location and intraventricular hemorrhage were 0.6144, 0,863, and 0.309, respectively.

**Table 2:** Characteristics of hematomas.

	Total (*n* = 125)	Conservative group (*n* = 74)	Surgical group (*n* = 51)	*p*
Hematoma volume (mL)	7.6 (6.0–9.6)	7.6 (5.5–9.5)	7.6 (6.5–9.7)	0.144
Location of hemorrhage				0.863
Unilateral tegmental	17 (13.6%)	10 (13.5%)	7 (13.7%)	
Bilateral tegmental	24 (19.2%)	15 (20.3%)	9 (17.6%)	
Basal-tegmental	39 (31.2%)	21 (28.4%)	18 (35.3%)	
Massive	45 (36%)	28 (37.8%)	17 (33.3%)	
Intraventricular hemorrhage(yes)	33 (26.4%)	22 (29.7%)	11 (21.6%)	0.309

### Outcomes of the total patient cohort

Table 3 provides the outcomes and complications in all patients and each in the conservative and surgical groups. The mortality of the conservative group at 6 months was 59/74 (79.7%) and that of the surgical group 9/51 (17.6%), and this difference was statistically significant (chi-square test, *p* < 0.001). Twenty-four patients (47.1%) achieved favorable outcomes (mRS < =4) in the surgical group, whereas this rate in the conservative group was only 5.4% (4/74); significant differences were observed between these two groups (chi-square test, p < 0.001). The most common complications after HBSH are central hyperthermia and stress ulcers. In this study, the incidence of central hyperthermia was 47.3% (35/74) and 47.3% (23/51) in the conservative and surgical groups, respectively, and that of stress ulcers was 50.0% (37/74) and 43.1% (22/51), respectively. However, the differences did not reach statistical significance between the two groups.

**Table 3:** Outcomes and complications of the total, conservative and surgical cohorts.

	Total (*n* = 125)	Conservative group (*n* = 74)	Surgical group (*n* = 51)	*p*
Mortality at 6 months	68 (54.4%)	59 (79.7%)	9 (17.6%)	<0.001
mRS at 6 months				<0.001
6	68 (54.4%)	59 (79.7%)	9 (17.6%)	
5	29 (23.2%)	11 (14.9%)	18 (35.3%)	
4	18 (14.4%)	4 (5.4%)	14 (27.5%)	
3	4 (3.2%)	0 (0%)	4 (7.8%)	
2	5 (4.0%)	0 (0%)	5 (9.8%)	
1	1 (0.8%)	0 (0%)	1 (2.0%)	
Complications				
Central hyperthermia	58 (46.4%)	35 (47.3%)	23 (45.1%)	0.809
Stress ulcers	59 (47.2%)	37 (50.0%)	22 (43.1%)	0.45

mRS: modified Rankin scale.

### Outcomes of surviving patients

In addition to comparing the overall outcomes of the two groups, the prognosis of surviving patients was also compared in the present study. Table 4 shows the outcomes and complications of surviving patients in the conservative and surgical groups. There was a significant difference in NIHSS scores at 6 months between the conservative and surgical groups [32 (IQR 28–34) vs. 26 (IQR 19–32), respectively; *p* = 0.032]. The median GCS score at 6 months for the conservative group was 7 [IQR 5–11], and that for the surgical group was 12 [IQR 7–15], and this difference was statistically significant (*p* = 0.043). In terms of mRS at 6 months in surviving patients, there was also a significantly higher percentage of favorable outcomes in the surgical group than in the conservative group (57.1% vs. 26.7%). However, no significant differences were observed in complications such as the incidence of central hyperthermia and stress ulcers between the two groups.

**Table 4:** Outcomes and complications of surviving patients.

	Surviving patients (*n* = 57)	Conservative group (*n* = 15)	Surgical group (*n* = 42)	*p*
NIHSS at 6 months	28 (24–33)	32 (28–34)	26 (19–32)	0.032
GCS at 6 months	9 (6–14)	7 (5–11)	12 (7–15)	0.043
mRS at 6 months				0.022
5	29 (50.9%)	11 (73.3%)	18 (42.9%)	
4	18 (31.6%)	4 (26.7%)	14 (33.3%)	
3	4 (7.0%)	0	4 (9.5%)	
2	5 (8.8%)	0	5 (11.9%)	
1	1 (1.8%)	0	1 (2.4%)	
Complications				
Central hyperthermia	20 (35.1%)	3 (20.0%)	17 (40.5%)	0.154
Stress ulcers	19 (33.3%)	2 (13.3%)	17 (40.5%)	0.056

GCS, Glasgow Score Scale; NIHSS, National Institutes of Health Stroke Scale; mRS, modified Rankin scale.

### Prognostic analysis in the surgical subgroup

We further analyzed the patients’ demographic and clinical characteristics in the surgical subgroup by dividing them into a favorable outcome group and an unfavorable outcome group to explore potential factors influencing the outcomes of patients undergoing surgery. All parameters are summarized in Table 5. Based on the definition of an unfavorable outcome in the present study, 27 patients (52.9%) had poor prognosis. Univariate analysis demonstrated significant differences between two groups for potential risk factors for poor 6-month prognosis after robot-assisted surgery for HBSH: initial GCS score [5 (IQR 4–7) vs. 3 (IQR 3–4), *p* < 0.001], NIHSS [36 (IQR 32–38) vs. 40 (IQR 38–40), *p* < 0.001], smoking history [45.8%(11/24) vs. 74.1%(20/27), *p* = 0.039], hematoma volume [6.9 (IQR 6.2–7.6) vs. 9.6 (IQR 7.3–11.4), *p* = 0.001], location of hemorrhage (*p* = 0.001).

**Table 5:** Univariate analysis in the surgical subgroup.

	Surgical patients (*n* = 51)	Favorable outcome (*n* = 24)	Unfavorable outcome (*n* = 27)	*p*
Age	55 (49–63)	54 (48.5–59.5)	58 (51–63)	0.241
Initial GCS score	4 (3–5)	5 (4–7)	3 (3–4)	<0.001
NIHSS on admission	38 (36–40)	36 (32–38)	40 (38–40)	<0.001
Sex				0.135
Male	35 (68.6%)	14 (58.3%)	21 (22.2%)	
Female	16 (31.4%)	10 (41.7%)	6 (77.8%)	
SBP on admission	172 (157–187)	172.5 (161–190)	172 (147.5–184.5)	0.433
DBP on admission	99 (92–107)	99 (94–106)	96 (88–107)	0.597
Medical history				
Smoking (yes)	31 (60.8%)	11 (45.8%)	20 (74.1%)	0.039
Diabetes (yes)	7 (13.7%)	1 (4.2%)	6 (22.2%)	0.061
Hyperlipidemia (yes)	9 (17.6%)	3 (12.5%)	6 (22.2%)	0.363
Medication before HBSH				
Anticoagulant drugs	1 (0.2%)	1 (4.2%)	0	0.284
Antiplatelet drugs	4 (7.8%)	0	4 (14.8%)	0.051
Irregular Antihypertensive Therapy(yes)	40 (78.4%)	20 (83.3%)	20 (74.1%)	0.422
Hematoma volume (mL)	7.6 (6.5–9.7)	6.9 (6.2–7.6)	9.6 (7.3–11.4)	0.001
Location of hemorrhage				0.001
Unilateral tegmental	7 (13.7%)	7 (29.2%)	0	
Bilateral tegmental	9 (17.6%)	7 (29.2%)	2 (7.4%)	
Basal-tegmental	18 (35.3%)	7 (29.2%)	11 (40.7%)	
Massive	17 (33.3%)	3 (12.5%)	14 (51.9%)	
Complications				
Central hyperthermia	23 (45.1%)	8 (33.3%)	15 (55.6%)	0.111
Stress ulcers	22 (43.1%)	7 (29.2%)	15 (55.6%)	0.058
Time from event to operation(h)	6 (3–12)	5 (3–15)	6 (4.5–12)	0.489

GCS, Glasgow Score Scale; NIHSS, National Institutes of Health Stroke Scale; mRS, modified Rankin scale.

## Discussion

The controversy regarding surgical treatment of brainstem hemorrhage is ongoing. Mangiardi et al. (31) indicated that HBSH is completely different from hemorrhage caused by brain stem cavernous malformation (BSCM); thus, surgical treatment for BSCM is not appropriate for HBSH. In 2015, “Guidelines for the Management of Spontaneous Intracranial Hemorrhage” formulated by the American Heart Association (AHA)/American Stroke Association (ASA) clearly indicated that surgical treatment for HBSH was not recommended (28). However, Ichimura et al. (18) reported an encouraging outcome in 5 HBSH patients of surgical management via the lateral or midline suboccipital and trans-rhomboid fossa approach. Currently, surgical treatments for HBSH is common in China (1, 15, 22, 32). The high incidence of HBSH in China as well as East Asia is one of the reasons; another reason is that the traditional Chinese family and clan culture in addition to medical insurance policies determine the surgical treatment decision by a patient’s relatives, even if they are informed of unfavorable outcomes of undergoing surgery.

Based on the advantages of the robot-assisted system, it has become the main surgical method for treatment of HBSH in our center. After performing more than 150 robotic surgeries annually, including SEEG, DBS, intracranial hematoma puncture drainage, intracranial lesion biopsy and intracranial abscess puncture drainage, we have gained our own experience with robot-assisted HBSH. During the phase of the preoperative projection, we usually reconstruct the three-dimensional morphology of the hematoma to design an individualized surgical projection. With image fusion of CTA, all visible blood vessels and sulci following the path can be avoided as much as possible to reduce vascular damage, which is of paramount importance for minimally invasive surgery (24, 33). At the registration stage, we usually adopt the minimum registration error method of bone markers to ensure the maximum accuracy of puncture. During the operation, the main factor affecting surgical accuracy is the slight displacement that may be caused by skull drilling. Therefore, after drilling the skull bone hole, we use one of the markers as the target for verification. Registration is conducted if the deviation exceeds 0.5 mm. When the puncture tube is inserted, it is recommended to move at a very slow speed, which should be approximately 1–2 mm per second. With our experience, no puncture bleeding or deviation has occurred during the robot-assisted intracranial hematoma drainage operations. This also indicates that the robot-assisted HBSH drainage operation is safe.

In our study, the prognosis of the surgical and conservative groups was compared first, which included mortality, mRS and complications. We observed an obvious trend, that is, the mortality rate of the surgical treatment was significantly lower than that of the conservative treatment (17.6% (9/51) vs. 79.7% (59/74), respectively), which is similar to relevant literature, regardless of whether patients received craniotomy or minimally invasive surgery. Lan et al. (20) performed microsurgery for 46 of 286 patients with HBSH over a 10-year study, and they observed a mortality rate of 30% in the surgical group compared with 70.4% in the conservative treatment group. In 2011, Jang et al. (6) reported a large case–control study of surgical procedures for HBSH from South Korea and a total of 86 patients who underwent stereotactic aspiration and microsurgery or simple external ventricular drainage, and they demonstrated that surgery did reduce the 30-day mortality rate. Yan et al. (22) performed stereotactic hematoma aspiration for 65 patients with HBSH using the modified Rankin Scale score (mRS) to assess the outcome status. They finally observed that the 30-day mortality rate after surgery was 23.1% (15 cases) and that the proportion of patients with encouraging neurological function at 90 days after surgery was 32.3% (21 cases), which indicated a promising result. We believe that removing a hematoma can reduce the local occupying effect of a hematoma and reduce release of hematoma decomposition products and vasoactive substances, which lead to sustained brain tissue damage and cause brain edema. If a penumbra exists in patients with HBSH, clot evacuation could also restore function to the surrounding brain tissue and improve outcome (34, 35). In addition, removing a hematoma can improve local blood flow (36, 37). Moreover, we sought to explore the timing of the operation. However, there is almost no literature demonstrating the timing of surgery for brainstem hemorrhage. In the present study, potential factors affecting the prognosis of surgical patients were also analyzed, but an association between the timing of the operation and prognosis was not observed. This may be caused by the fact that all operations in our center were performed within 72 h of onset. Nonetheless, based on the experience of some literature regarding intracranial hematoma operation, we recommended that the operation should be performed as early as possible within 72 h (21, 38). In general, we both patients’ family members and neurologists are concerned with whether the patient is more likely to survive after surgical procedures. Other critical questions are whether the survival rate of patients indeed improves after surgery and quality of life.

In comparison of mRS at 6 months after operation, we observed significantly better outcomes in the surgical group than the conservative group (*p* < 0.001). Next, in comparison of the prognosis in surviving patients, we found that the surgical group was still at an advantage over the conservative treatment group in terms of NIHSS score, GCS score and mRS scores after 6 months. However, we also observed that the difference in mRS was not as obvious as in the previous comparison (*p* = 0.022), which means that the operation improved the survival rate but may have caused more disabilities. According to Youchao et al. (39), the proportion of patients with encouraging neurologic function after microsurgical craniotomy was 15.6%. Lan et al. (20) found a 32.6% severe disability rate after microsurgery in 46 patients with HBSH. They postulated that the surgical group presented more neurological deficits than the conservative group because of their prolonged survival time. Li et al. also observed a similar result: the proportion of patients with good neurological recovery at 90 days after the operation was only 32.3% (21/65) (22). A similar situation has been reported in literature about intracranial hemorrhage (40). In fact, not all patients with HBSH can benefit from surgery, which should be performed in selected cases (15, 22, 41). Consequently, we analyzed the patients in the surgical subgroup and observed differences in parameters in those with different prognoses to explore factors affecting the prognosis of patients who undergo surgery.

In the surgical subgroup, the proportion of patients with favorable outcomes at 6 months was 47.1% (24/51). Notably, the reason for the prognostic grouping (an unfavorable outcome was defined as an mRS score of 5 and 6) is that we found in clinical practice in China that even a severe disability status is still more acceptable by the majority of relatives than a vegetative state or death with severe diseases such as HBSH. Usually, the lifestyle of three or more generations living together in our country will allow family members to take care of patients conveniently. In univariate analysis of surgical prognosis in the present study, we observed that the initial GCS score, NIHSS score, hematoma volume, and location of hemorrhage were potential predictors of poor 6-month prognosis after surgery. It is generally recognized that a low GCS score is associated with a greater hematoma volume and a high NIHSS score, especially in patients with conservative treatment (7). Toshinari et al. (42) found that a GCS score less than 6 was one of the independent factors influencing 30-day mortality in 101 patients with pontine hemorrhage treated conservatively. After evaluating the mortality rate of pontine hemorrhage and establishing the factors related to the outcomes, Ye et al. (43) demonstrated that a low GCS score and a large hematoma volume (>4 mL) may be related to an unreasonable 3-year prognosis. Jang et al. (6) reported that consciousness and a hematoma volume of <5 mL were associated with 90-day functional recovery. Notably, in our study, most of the patients who were in deep coma (GCS < 5) after massive brain stem hemorrhage were given surgery at an early stage (< 12 h). However, we found that even if the hematoma was removed quickly, their prognosis could not be improved. We believe that the primary injury, which is the swelling and destruction of the brain stem by the hematoma, plays a dominant role after HBSH, and even if the secondary injury is relieved immediately, it is difficult to change the outcome in the later stage of neuroplasty. Intriguingly, we found another phenomenon in which the threshold of hematoma volume that led to poor prognosis was higher than that of conservative treatment, which is usually 4–5 mL (43, 44). It was also mentioned in a study by Chen et al. that an amount of hematoma exceeding 10 mL is a factor of poor prognosis after surgery (15). Notably, some patients with moderate bleeding may benefit from surgery; furthermore, the surgical prognosis may be anticipated in terms of the hematoma volume before the operation.

This study also shows that different types of hematoma lead to different prognoses after surgery. Similar results have been reported in the literature (22, 39). The prognosis of unilateral and bilateral tegmental types is usually better than that of other types of hematoma. A reasonable explanation is that the bleeding of the tegmentum usually derives from a smaller-caliber lateral arteriole, which generally causes a smaller hematoma (43, 45). In addition, a hematoma of the tegmental part has a less primary destructive effect on the ascending activation system, life-sustaining nuclei, and pyramidal tract of the brain stem than other types, as well as reduced brain stem swelling (22).

## Limitations

The results of the present study in patients with HBSH who underwent robot-assisted drainage of brain stem hematoma will be useful for clinical practice. However, the limitations of this study must be noted. Although a relatively large sample of patients was included, selection bias is unavoidable in a retrospective study. Another limitation is that due to the limitation of sample size, we only conducted univariate analysis without multivariate analysis in prognosis comparison of surgical patients. However, as of the completion of this manuscript, we have completed more than 50 robot-assisted operations in patients with HBSH this year. In a follow-up study, we will conduct multivariate analysis to explore independent predictors that affect the prognosis after surgery and further evaluate the prediction model for prognosis in patients with HBSH after robot-assisted surgery. In addition, our two groups of patients included a small number of patients with occlusive hydrocephalus. Although the outcomes of these patients did not affect the statistical results of this study, they may have a significant impact as a potential statistical bias factor when the sample size is expanded in the future. Therefore, we will pay close attention to this confounding factor in future studies.

## Conclusion

Patients with HBSH may benefit from robot-assisted minimally invasive drainage, which is a safe, simple and brief surgical procedure. Based on the results of the present study, robot-assisted minimally invasive drainage of brain stem hematoma may significantly reduce mortality and improve prognosis. The authors also concluded that surgery should be administered to selected patients.

## Data availability statement

The raw data supporting the conclusions of this article will be made available by the authors, without undue reservation.

## Ethics statement

The studies involving humans were approved by Ganzhou People’s Hospital ethics committee. The studies were conducted in accordance with the local legislation and institutional requirements. Written informed consent for participation in this study was provided by the participants' legal guardians/next of kin. Written informed consent was obtained from the individual(s) for the publication of any potentially identifiable images or data included in this article.

## Author contributions

ZT: Conceptualization, Funding acquisition, Writing – original draft, Writing – review & editing. WH: Conceptualization, Writing – review & editing. QC: Data curation, Writing – review & editing. CG: Data curation, Writing – review & editing. KZ: Data curation, Writing – review & editing. WW: Data curation, Writing – review & editing. QJ: Conceptualization, Writing – original draft, Writing – review & editing. RY: Writing – review & editing.
